# Meta-analysis of Percutaneous vs. Surgical Approaches Radiofrequency Ablation in Hepatocellular Carcinoma

**DOI:** 10.3389/fsurg.2021.788771

**Published:** 2022-01-04

**Authors:** Xiaozhun Huang, Yibin Liu, Lin Xu, Teng Ma, Xin Yin, Zhangkan Huang, Caibin Wang, Zhen Huang, Xinyu Bi, Xu Che

**Affiliations:** ^1^Department of Hepatobiliary Surgery, National Cancer Center/National Clinical Research Center for Cancer/Cancer Hospital and Shenzhen Hospital, Chinese Academy of Medical Sciences and Peking Union Medical College, Shenzhen, China; ^2^Department of General Surgery, Longgang District Central Hospital of Shenzhen, Shenzhen, China; ^3^Department of Hepatobiliary Surgery, National Cancer Center/National Clinical Research Center for Cancer/Cancer Hospital, Chinese Academy of Medical Sciences and Peking Union Medical College, Beijing, China; ^4^Department of Gastrointestinal and Pancreatic Surgery, National Cancer Center/National Clinical Research Center for Cancer/Cancer Hospital, Chinese Academy of Medical Sciences and Peking Union Medical College, Beijing, China

**Keywords:** radiofrequency ablation, hepatocellular carcinoma, surgical, percutaneous, meta-analysis

## Abstract

**Background:** Radiofrequency ablation (RFA) is a curative modality for hepatocellular carcinoma (HCC) patients who are not suitable for resection. It remains controversial whether a surgical or percutaneous approach is more appropriate for HCC.

**Method:** A search was performed on the PubMed, Web of Science, Embase, and Cochrane Library databases from the date of database inception until April 17, 2021. Studies reporting outcomes of comparisons between surgical RFA (SRFA) and percutaneous RFA (PRFA) were included in this study. The meta-analysis was performed using the Review Manager 5.3 and Stata 12.0 software.

**Result:** A total of 10 retrospective studies containing 12 cohorts, involving 740 patients in the PRFA group and 512 patients in the SRFA group, were selected. Although the tumor size in PRFA group was smaller than the SRFA group (*p* = 0.007), there was no significant difference in complete ablation rate between the SRFA and PRFA groups (95.63% and 97.33%, respectively; Odds ratio [OR], 0.56; 95% confidence intervals [CI], 0.26–1.24; *p* = 0.15). However, the SRFA group showed a significantly lower local tumor recurrence than the PRFA group in the sensitivity analysis (28.7% in the PRFA group and 21.79% in the SRFA group, respectively; OR, 1.84; 95% CI, 1.14–2.95; *p* = 0.01). Pooled analysis data showed that the rate of severe perioperative complications did not differ significantly between the PRFA and SRFA groups (14.28% and 12.11%, respectively; OR, 1.30; 95% CI, 0.67-2.53; *p* = 0.44). There was no significant difference in the 1-, 3-, and 5-year overall survival rates, as well as the 1- and 3-year disease-free survival (DFS) between the PRFA and SRFA groups. The 5-year DFS of the PRFA group was significantly lower than the SRFA group (hazard ratio 0.73; 95% CI 0.54–0.99).

**Conclusion:** Based on our meta-analysis, the surgical route was superior to PRFA in terms of local control rate. Furthermore, the surgical approach did not increase the risk of major complications.

## Introduction

Radiofrequency ablation (RFA) is recognized as a curative modality for early-stage hepatocellular carcinoma (HCC), especially in patients who are not suitable for resection and liver transplantation (1–4). Although RFA for HCC can be performed using percutaneous or surgical approaches, a percutaneous approach using external ultrasonic (ETUS) is the least invasive method, with a low cost and short hospital stay (5). However, percutaneous radiofrequency ablation (PRFA) is associated with lower accuracy in cancer staging, poor accessibility in certain areas of the liver, can damage or perforate adjacent visceral organs, and cause diaphragmatic injury (6–8). These issues can be addressed using surgical radiofrequency ablation (SRFA), which is performed with open and laparoscopic approaches utilizing an intraoperative ultrasonic (IOUS) probe. It is considered a more accurate and effective method for HCC that develops in relatively inaccessible areas (9). At present, only a few studies have examined the advantages and disadvantages of percutaneous and surgical approaches. Whether SRFA is more appropriate for patients with HCC compared to PRFA remains unclear and is up for debate (10–12).

In this study, we performed a meta-analysis with inclusion and exclusion criteria to review the currently available published data comparing the safety and efficacy of these two therapeutic approaches in patients with HCC.

## Materials and Methods

The present meta-analysis was performed according to the criteria defined by the Preferred Reporting Items for Systematic Reviews and Meta-analyses statement (13).

### Data Source and Search Strategy

A literature search on the PubMed, Web of Science, Embase, and Cochrane Library databases was performed to select relevant articles with no restrictions on regions starting from the date of database inception until April 17, 2021. The following keywords were searched in titles and abstracts: (hepatocellular carcinoma) AND [(radiofrequency) OR (ablation)] AND {[(open) OR (surgery) OR (laparoscopic) OR (surgical) OR (laparoscopy)] AND (percutaneous)}. All the retrieved articles were reviewed, with their reference lists manually screened to identify additional studies. When multiple reports described the same patient population, the most recent or complete report was included. The literature search was independently conducted by two researchers, and any disagreements were resolved by the adjudicating senior authors.

### Inclusion and Exclusion Criteria

Studies were included in the meta-analysis when both of the following inclusion criteria were met: 1. Comparisons of postoperative and survival outcomes between PRFA and SRFA. 2. Confirmation of HCC in study patients based on clinical diagnosis. The exclusion criteria were as follows: 1. Lack of reporting or inability to calculate relevant outcomes based on available data. 2. Non-human experimental study design. 3. Publication types, other than randomized controlled trials and observational studies, such as editorials, letters to the editor, review articles, and case reports.

### Data Extraction and Study Outcomes

After the removal of duplicates, the titles and abstracts of the retrieved studies were screened and sequentially excluded according to the eligibility criteria. In the event of any uncertainties after screening the titles and abstracts, the complete text was independently assessed by two investigators, and any discrepancies were resolved by consensus. The primary outcomes were complete ablation rate, postoperative complication, and recurrence rates.

### Quality Assessment and Statistical Analysis

The completeness, plausibility, and integrity of the available data were reviewed before being compiled into a single database. The methodological quality of retrospective studies was assessed using the modified Newcastle–Ottawa scale (mNOS) (14, 15), which comprised three factors: patient selection, comparability of study groups, and outcome assessment. Each study was given stars based on a score of 0–9, with studies receiving eight or more stars considered as high quality. Any discrepancies were resolved by consensus. The meta-analysis was performed using the Review Manager 5.3 software (Cochrane Collaboration, Oxford, UK). The weighted mean difference (WMD) and odds ratio (OR) were used to compare continuous and dichotomous variables. All results were reported with 95% confidence intervals (CI). Statistical heterogeneity among the included studies was assessed using the chi-squared test, with a *p* < 0.05 considered significant, and heterogeneity was quantified using the *I*^2^ statistic. In the event of significant heterogeneity among the included studies, the random-effects model was used for pooled analyses; otherwise, the fixed-effects model was used (16). Publication bias was examined using the Stata 12.0 software (Stata Corporation, College Station, TX, USA).

## Results

### Search Results

**Figure 1:**
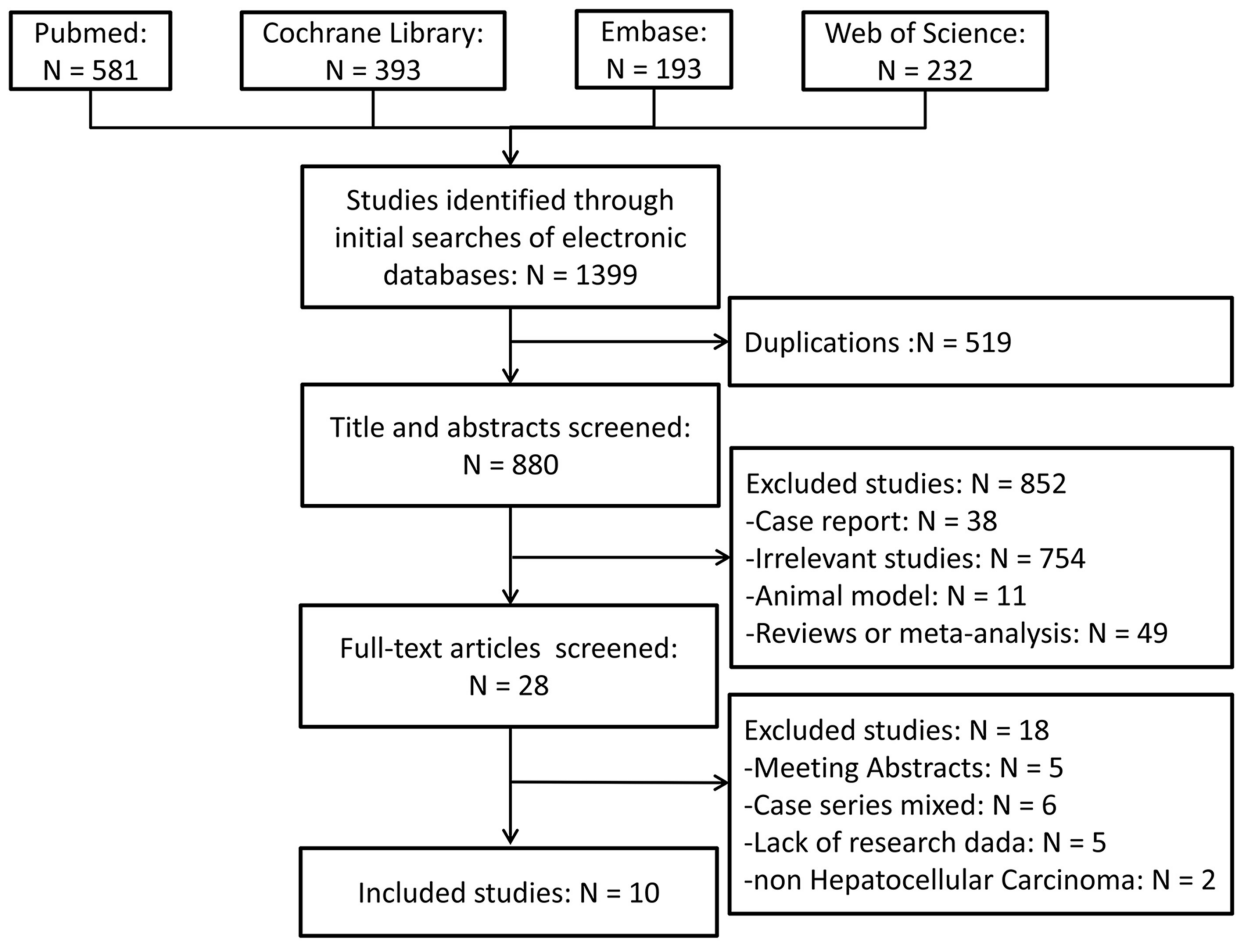
Schematic illustration of the literature search and study-selection criteria.

The literature search and study selection criteria are schematically illustrated in Figure 1. A total of 1,399 publications were retrieved following an initial search on the biomedical databases. After reviewing the titles and/or abstracts, 519 articles were eliminated because of duplication, and 852 articles were excluded because they were deemed irrelevant for the present study. Full texts of 28 studies were reviewed: five were available as abstracts only, six were case series with inappropriate control groups, two had samples mixed with other liver malignancies, and five lacked research data. The remaining ten studies (9–12, 17–22) that evaluated the outcomes of patients with HCC using different approaches were included in the meta-analysis. Manual screening of the reference lists of these ten publications identified no additional studies. The two reviewers were in complete agreement for both the study selection and the quality assessment of trials. Two studies (11, 20) contained two sets of readily available independent data, which were extracted and analyzed separately.

### Characteristics of the Included Studies

The characteristics of the ten studies (9–12, 17–22) included in the meta-analysis are summarized in Table 1. All studies were published between 2000 and 2018. The sample size in individual studies ranged from 60 to 172, for a total of 1,252 patients, consisting of 740 (59.11%) and 512 (40.89%) patients with PRFA and SRFA, respectively. The tumor size in the PRFA group was smaller than in the SRFA group (*p* = 0.007; Supplementary Figure S1). There was no significant difference in Child-Pugh A between the two groups (*p* = 0.13; Supplementary Figure S2), and the rate of chronic hepatitis B virus infection was indifferent (*p* = 0.33; Supplementary Figure S3). All patients in three studies (12, 17, 21) had a core biopsy of the liver cirrhosis, and 3 studies (10, 19, 20) indicated part of patients underlying cirrhosis. Two studies (9, 20) described surgical approach was offered in the dangerous circumstances: Percutaneous ablation might lead to pneumothorax or damage to the diaphragm; or tumors located near the visceral organs such as the gallbladder, colon, or stomach.

**Table 1:** Summary of characteristics of included studies.

**Study**	**Location/year**	**Surgical**	**Number of**		**Number of**		**Childs-Pugh**		**Tumor size**		**Tumor number**	
		**approach**	**patients**		**nodules**		**(A: B: C)**		**(cm)**		**(solitary: multiple)**	
			**PRFA**	**SRFA**	**PRFA**	**SRFA**	**PRFA**	**SRFA**	**PRFA**	**SRFA**	**PRFA**	**SRFA**
Eun, H. S	Korea/2017	laparoscopic	33	33	36	40	NR	NR	1.7	1.7	31:2	27:6
Chen, S	China/2018	laparoscopic	30	30	32	46	NR	NR	NR	NR	NR	NR
Khan, M. R (1)	China/2007	laparoscopic + open	92	63	110	81	87:5:0	54:8:1	1.9 ± 0.6	2.2 ± 0.6	75:17	51:12
Khan, M. R (2)	China/2007	laparoscopic + open	25	48	27	63	22:3:0	45:2:1	3.6 ± 0.4	3.9 ± 0.5	23:2	37:11
Li, J	China/2018	open	54	35	71	48	49:4:0	34:2:0	2.8 (0.8–6.0)	3.5 (0.4–5.0)	NR	NR
Hirooka, M	Japan/2009	laparoscopic	37	37	44	42	29:8:0	25:12:0	2.49 ± 0.46	2.6 ± 0.69	NR	NR
Sherif, Z	Egypt/2008	laparoscopic	30	30	36	34	6:24:0	10:20:0	NR	NR	22:8	10:20
Zhang, W (1)	China/2016	laparoscopic	77	19	175	42	74:3:0	18:1:0	2.3 ± 0.6	2.3 ± 0.6	NR	NR
Zhang, W (2)	China/2016	open	77	58	175	137	74:3:0	57:1:0	2.3 ± 0.6	2.3 ± 0.5	NR	NR
Curley, S. A	Italy/2000	laparoscopic + open	76	34	84	65	17:30:29	33:1:0	2.8 ± 0.8	4.6 ± 1.7	NR	NR
Raut, C. P	American/2005	open	140	32	190	NR	59:46:35	NR	3	NR	101:39	NR
Huang, J. W	China/2011	open	69	93	89	108	61:8:0	93:0:0	1.7 ± 1.1	1.8 ± 1.0	51:18	79:14

*PRFA, percutaneous radiofrequency ablation; SRFA, surgical radiofrequency ablation; NR, not reported*.

### The Methodological Quality of the Included Studies

Studies were evaluated for sources of bias using the mNOS. In general, the quality of all included studies was moderate (Table 2). Among the ten studies, four (17, 19, 20, 22) achieved a score of 9/9, four (10–12, 21) scored 8/9, and two (9, 18) scored 7/9. Eight studies (10–12, 17, 19–22) indicated the follow-up duration, and all studies provided intraoperative and postoperative outcomes. The methods for handling missing data were adequately described in one study (17).

**Table 2:** Risk of bias using the modified Newcastle-Ottawa Scale.

**Study**	**Selection**				**Comparability**	**Outcome**			**Score**
	**Representativeness of exposed cohort**	**Selection of non-exposed cohort**	**Exposure**	**Outcome of interest not present at start**	**Comparability of PRFA vs. SRFA**	**Assessment of outcome**	**Follow-up**	**Adequacy of follow-up**	
Eun, H. S	Truly representative	Same	Surgical records	Yes	Restricted, matched	Record linkage	Yes	Complete	9⋆
Chen, S	Truly representative	Same	Surgical records	Yes	Unclear	Record linkage	Yes	Unclear	7⋆
Khan, M. R	Truly representative	Same	Surgical records	Yes	Restricted, matched	Record linkage	Yes	Complete	9⋆
Li, J	Truly representative	Same	Surgical records	Yes	Restricted, matched	Record linkage	Yes	Unclear	8⋆
Hirooka, M	Truly representative	Same	Surgical records	Yes	Not restricted, matched	Record linkage	Yes	Unclear	7⋆
Sherif, Z	Truly representative	Same	Surgical records	Yes	Restricted, matched	Record linkage	Yes	Complete	9⋆
Zhang, W	Truly representative	Same	Surgical records	Yes	Restrictions, matched	Record linkage	Yes	Complete	8⋆
Curley, S. A	Truly representative	Same	Surgical records	Yes	Unclear	Record linkage	Yes	Complete	8⋆
Raut, C. P	Truly representative	Same	Surgical records	Yes	Restrictions, matched	Record linkage	Yes	Complete	8⋆
Huang, J. W	Truly representative	Same	Surgical records	Yes	Restrictions, matched	Record linkage	Yes	Complete	9⋆

*PRFA, percutaneous radiofrequency ablation; SRFA, surgical radiofrequency ablation*.

### Primary Outcomes

#### Complete Ablation Rate

**Figure 2:**
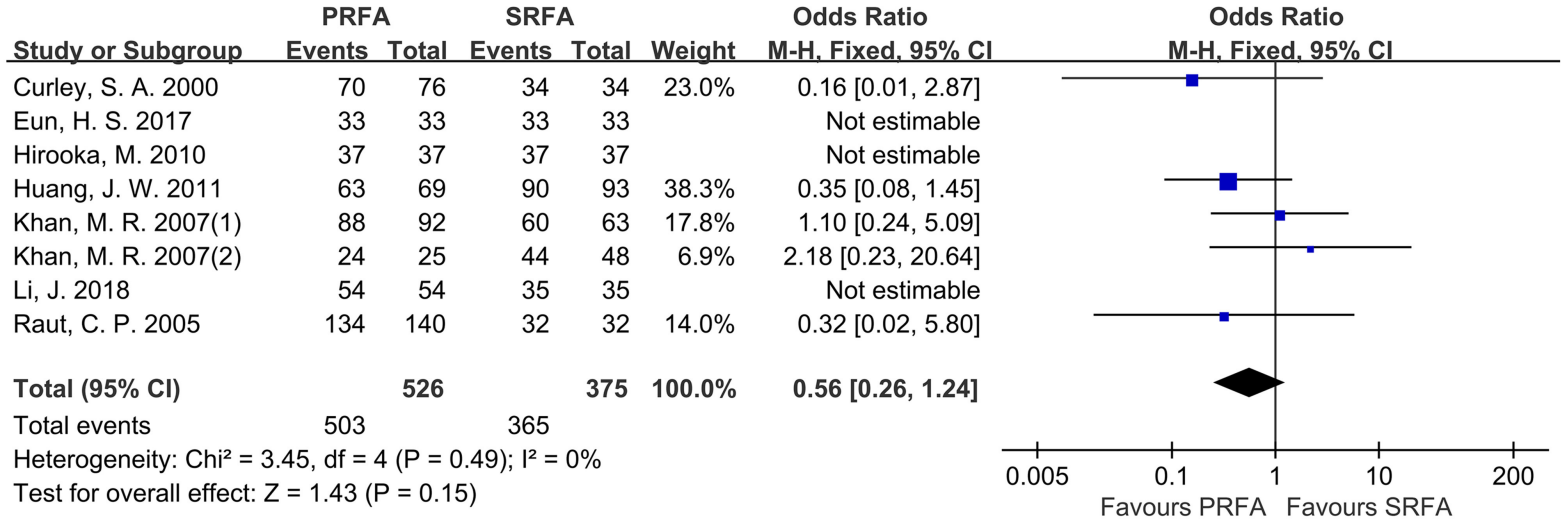
Forest plots for complete ablation rate. Forest plot for complete ablation rate indicates no significantly between the PRFA group as compared with that in the SRFA group (95.63% and 97.33%, respectively; OR, 0.56; 95%CI, 0.26-1.24; *p* = 0.15), and no statistical heterogeneity was found (*χ*^2^ = 3.45; *p* = 0.49, *I*^2^ = 0%).

When data from all included trials were pooled, seven studies (10, 12, 17–21) reported a complete ablation rate. Although the PRFA group appeared to have a lower complete ablation rate than the SRFA group, a meta-analysis using the fixed effects model revealed no significant difference in complete ablation rate between the two groups (95.63 and 97.33%, respectively; OR, 0.56; 95%CI, 0.26–1.24; *p* = 0.15), as well as no statistical heterogeneity (*χ*^2^ = 3.45; *p* = 0.49, *I*^2^ = 0%; Figure 2).

#### Tumor Recurrence

**Figure 3:**
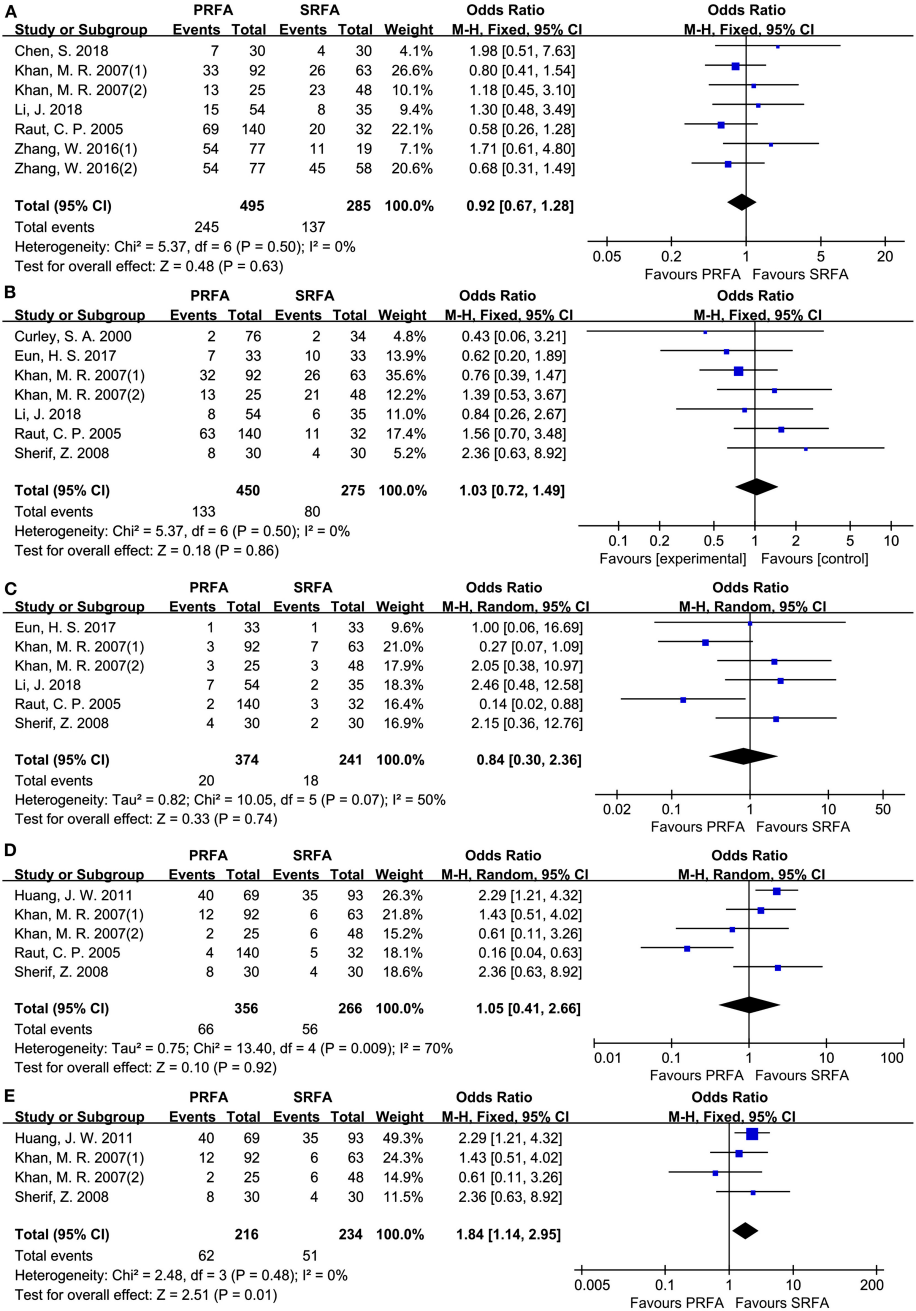
Forest plot for tumor recurrence. **(A)** Forest plot for total recurrence indicates no significantly between the PRFA group and the SRFA group (49.49% and 48.07%, respectively; OR, 0.92; 95% CI, 0.67-1.28; *p* = 0.63). **(B)** Forest plot for intrahepatic recurrence indicates no significantly between PRFA and SRFA (29.56% and 29.09%, respectively; OR, 1.03; 95% CI, 0.72-1.49; *p* = 0.86). **(C)** Forest plot for extrahepatic metastasis indicates no significantly between PRFA and SRFA (5.35% and 7.47%, respectively; OR, 0.84; 95% CI, 0.30-2.36; *p* = 0.61). **(D)** Forest plot for local recurrence indicates no significantly between PRFA and SRFA (18.54% and 21.05%, respectively; OR, 1.05; 95% CI, 0.41-2.66; *p* = 0.92; *χ*^2^ = 13.4; *p* = 0.009, *I*^2^ = 70%). **(E)** Forest plot for sensitivity analysis of local recurrence indicates less recurrent in the SRFA group (28.7% and 21.79%, respectively; OR, 1.84; 95% CI, 1.14-2.95; *p* = 0.01; *χ*^2^ = 2.48; *p* = 0.48, *I*^2^ = 0%).

A total of seven cohorts (9–11, 20, 21) were evaluated, with 780 patients reporting overall recurrence data. According to a meta-analysis, the total recurrence in PRFA did not significantly differ from SRFA (49.49 and 48.07%, respectively; OR, 0.92; 95 CI, 0.67–1.28; *p* = 0.63; Figure 3A). In addition, the rate of intrahepatic recurrence between the PRFA and SRFA groups was not significantly different (29.56 and 29.09%, respectively; OR, 1.03; 95% CI, 0.72–1.49; *p* = 0.86; Figure 3B), and there was no statistical heterogeneity (*χ*^2^ = 5.37; *p* = 0.50, *I*^2^ = 0%). Similarly, the rate of extrahepatic metastasis in the PRFA group was not significantly different compared to the SRFA group (5.35 and 7.47%, respectively; OR, 0.84; 95% CI, 0.30–2.36; *p* = 0.61; Figure 3C). However, there was moderate heterogeneity in the data reported by the included studies (*χ*^2^ = 10.05; *p* = 0.07, *I*^2^ = 50%). The indifferent rate of complete ablation resulted in no significant difference in the rates of local recurrence between the PRFA and SRFA groups (18.54 and 21.05%, respectively; OR, 1.05; 95% CI, 0.41–2.66; *p* = 0.92; Figure 3D), and statistical heterogeneity was moderate (*χ*^2^ = 13.40; *p* = 0.009, *I*^2^ = 70%). A sensitivity analysis showed that there was significantly less recurrence in the SRFA group (28.7% in PRFA and 21.79% in SRFA, respectively; OR, 1.84; 95% CI, 1.14–2.95; *p* = 0.01; Figure 3E).

### Postoperative Outcomes

**Figure 4:**
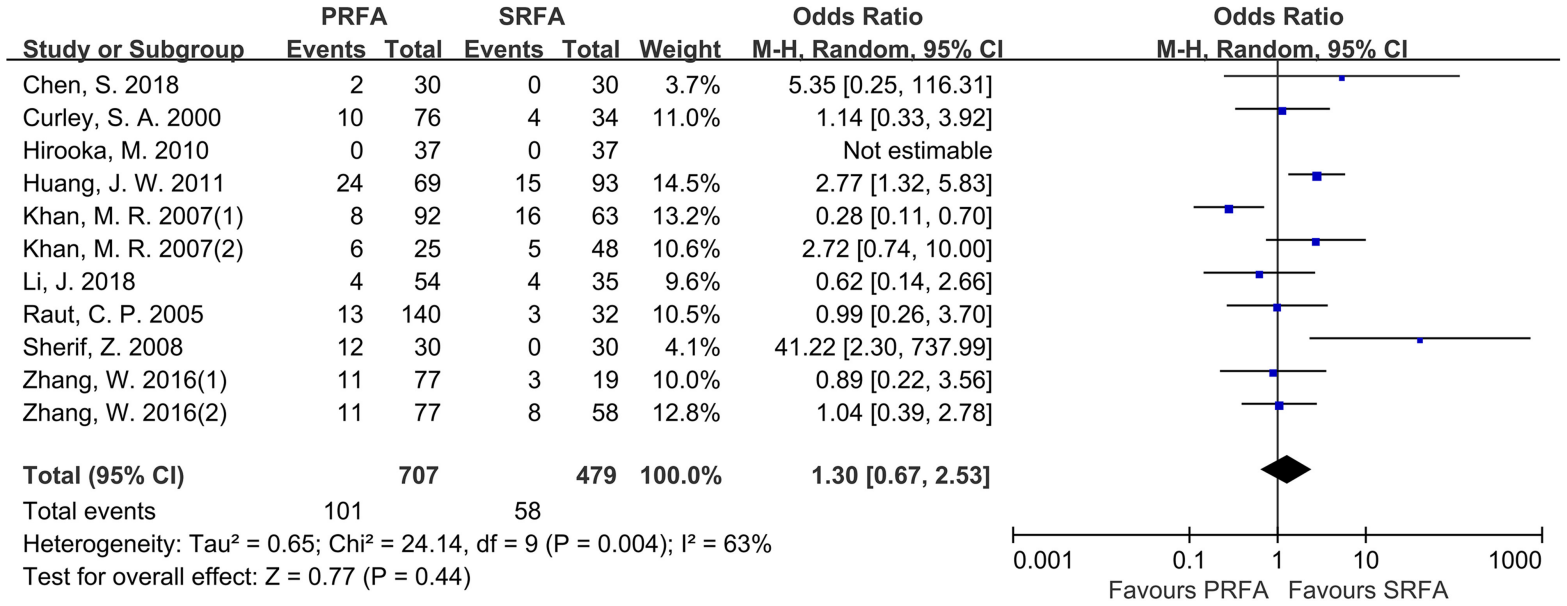
Forest plot for severe perioperative complications. Forest plot for the rate of severe perioperative complications indicates no significantly between the PRFA group and the SRFA group (14.28% and 12.11%, respectively; OR, 1.30; 95% CI, 0.67-2.53; *p* = 0.44).

According to pooled analysis data from 11 cohorts of nine included studies (9–12, 18–22), there was no significant difference in the rate of severe perioperative complications between the PRFA and SRFA groups (14.28 and 12.11%, respectively; OR, 1.30; 95% CI, 0.67–2.53; *p* = 0.44; Figure 4). However, there was a significant degree of heterogeneity in the data reported by the included studies (*χ*^2^ = 24.14; *p* = 0.004, *I*^2^ = 63%).

Among the various treatment-related complication (Table 3), internal hemorrhage rate appeared to be higher in the PRFA group, but meta-analysis revealed that it was not significantly different compared with the SRFA group. There was no difference in the rate of biliary injury, liver abscess, intestinal complications, pain, fever, arrhythmia atelectasis/hydropneumothorax, pleural effusion, and organ failure, including hepatic and renal failure, between the PRFA and SRFA groups. The rate of ascites in the PRFA group was significantly lower compared to the SRFA group (3.58% and 7.92%, respectively; OR, 0.57; 95% CI, 0.33–0.99; *p* = 0.05;), and there was heterogeneity in the data reported by the included studies (*χ*^2^ = 11.72; *p* = 0.23, *I*^2^ = 23%). Three cohorts reported that skin burn was higher in the PRFA group than in the SRFA group, with a meta-analysis showing that it was statistically significant (3.24 and 0%, respectively; OR, 5.30; 95% CI, 1.12–25.05; *p* = 0.04).

**Table 3:** Meta-analysis results of all available studies in complication treatment related.

**Postoperative outcomes**	**No. Cohorts**	**No. Patients**		**Heterogeneity test**		**Model**	**OR**	**95%CI**	***P***
		**PRFA**	**SRFA**	***I*** **^2^**	***P***				
Internal hemorrhage	7	499	296	23	0.26	Fixed	1.73	0.80–3.73	0.16
Biliary injury	5	278	248	0	0.47	Fixed	1.54	0.53–4.42	0.42
Pain	5	402	270	38	0.17	Random	0.60	0.16–2.28	0.46
Liver abscess	4	278	248	0	0.52	Fixed	1.17	0.44–3.13	0.75
Ascites	10	670	442	23	0.23	Fixed	0.57	0.33–0.99	0.05
Organ failure	4	223	175	47	0.13	Random	1.25	0.14–10.95	0.84
Intestinal complications	3	246	140	0	0.48	Fixed	1.05	0.20–5.56	0.95
Fever	3	163	160	0	0.78	Fixed	0.57	0.31–1.05	0.07
Arrhythmia	5	387	212	9	0.41	Fixed	0.54	0.17–1.78	0.31
Atelectasis/Hydropneumothorax	4	333	177	0	0.89	Fixed	0.59	0.13–2.69	0.50
Pleural effusion	10	670	442	0	0.53	Fixed	0.61	0.33–1.09	0.10
Skin burn	4	216	234	0	0.91	Fixed	5.30	1.12–25.05	0.04

*PRFA, percutaneous radiofrequency ablation; SRFA, surgical radiofrequency ablation; OR, Odds ratio; 95%CI, 95% confidence intervals*.

**Figure 5:**
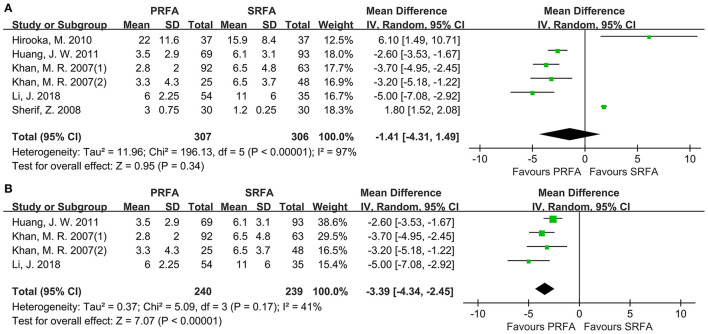
Forest plot for length of hospital stay. **(A)** Forest plot for length of hospital stay indicates no significantly between the PRFA group and the SRFA group (WMD, 0.61 days longer in the SRFA group; 95% CI, −3.28–2.06; *p* = 0.65; (*χ*^2^ = 161.19; *p* < 0.00001, *I*^2^ = 97%). **(B)** Forest plot for sensitivity analysis of length of hospital between PRFA and open approach showed that PRFA group had a significant reduced hospital duration (WMD, 1.4 days longer in the SRFA group; 95% CI, −4.34 to −2.45; *p* < 0.00001; *χ*^2^ = 5.09; *p* = 0.17, *I*^2^ = 41%).

The length of hospital stay was reported by six cohorts. Although the PRFA group had a shorter hospital stay, a meta-analysis using the random-effects model found no significant difference (WMD, 1.41 days longer in the SRFA group; 95% CI, −4.31 to 1.49; *p* = 0.34; Figure 5A). However, there was statistical heterogeneity (*χ*^2^ = 196.13; *p* < 0.00001, *I*^2^ = 97%). A subgroup analysis revealed that the PRFA group had a significantly reduced hospital duration compared to the open approach group (WMD, 3.39 days longer in the SRFA group; 95% CI, −4.34 to −2.45; *p* < 0.00001; *χ*^2^ = 5.09; *p* = 0.17, *I*^2^ = 41%, Figure 5B).

### Survival Analysis

**Figure 6:**
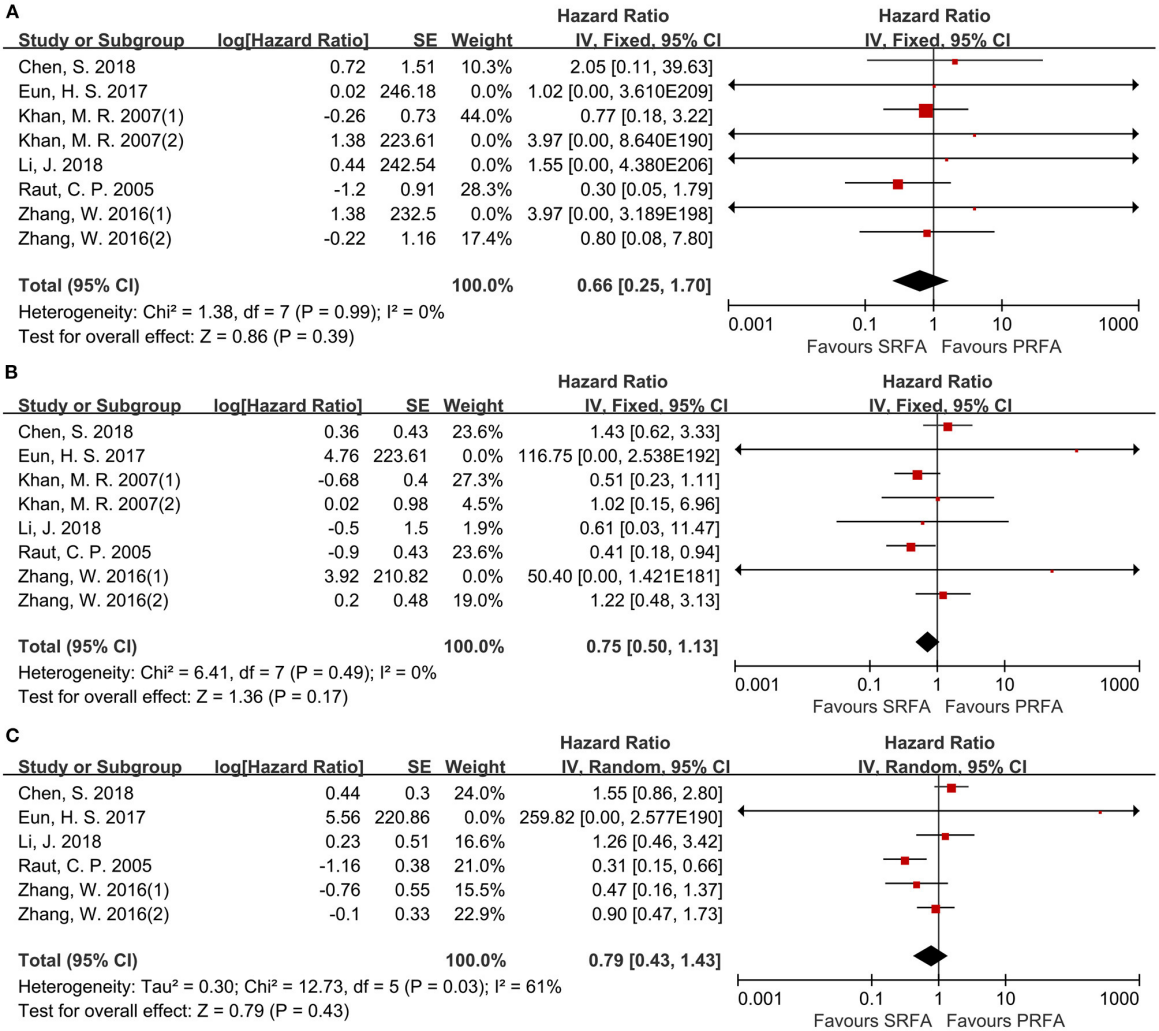
Forest plot for OS. **(A)** Forest plot for 1-year OS indicates no significantly between the PRFA group and the SRFA group (HR, 0.66; 95% CI: 0.25–1.70). **(B)** Forest plot for 3-year OS indicates no significantly between the PRFA group and the SRFA group (HR, 0.75; 95% CI: 0.50–1.13). **(C)** Forest plot for 5-year OS indicates no significantly between the PRFA group and the SRFA group (HR, 0.79; 95% CI: 0.43–1.43)

Eight cohorts (9–11, 17, 20, 21) reported the 1- and 3-year overall survival (OS) rates and six cohorts (9–11, 17, 21) reported the 5-year OS rates for the PRFA and SRFA groups using hazard ratios (HR). A meta-analysis revealed that there were no significant differences in 1-, 3-, and 5-year OS rates between the PRFA and SRFA groups (Figures 6A–C). The HR for the 1-, 3-, and 5-year OS rates were 0.66 (95% CI 0.25–1.70), 0.75 (95% CI 0.50–1.13), and 0.79 (95% CI 0.43–1.43), respectively. The data for the 5-year timepoints revealed moderate heterogeneity (*I*^2^ = 61%, *p* = 0.03), whereas the 1- and 3-year OS rates showed no heterogeneity (*I*^2^ = 0%, *p* = 0.99 and *I*^2^ = 0%, *p* = 0.49, respectively).

**Figure 7:**
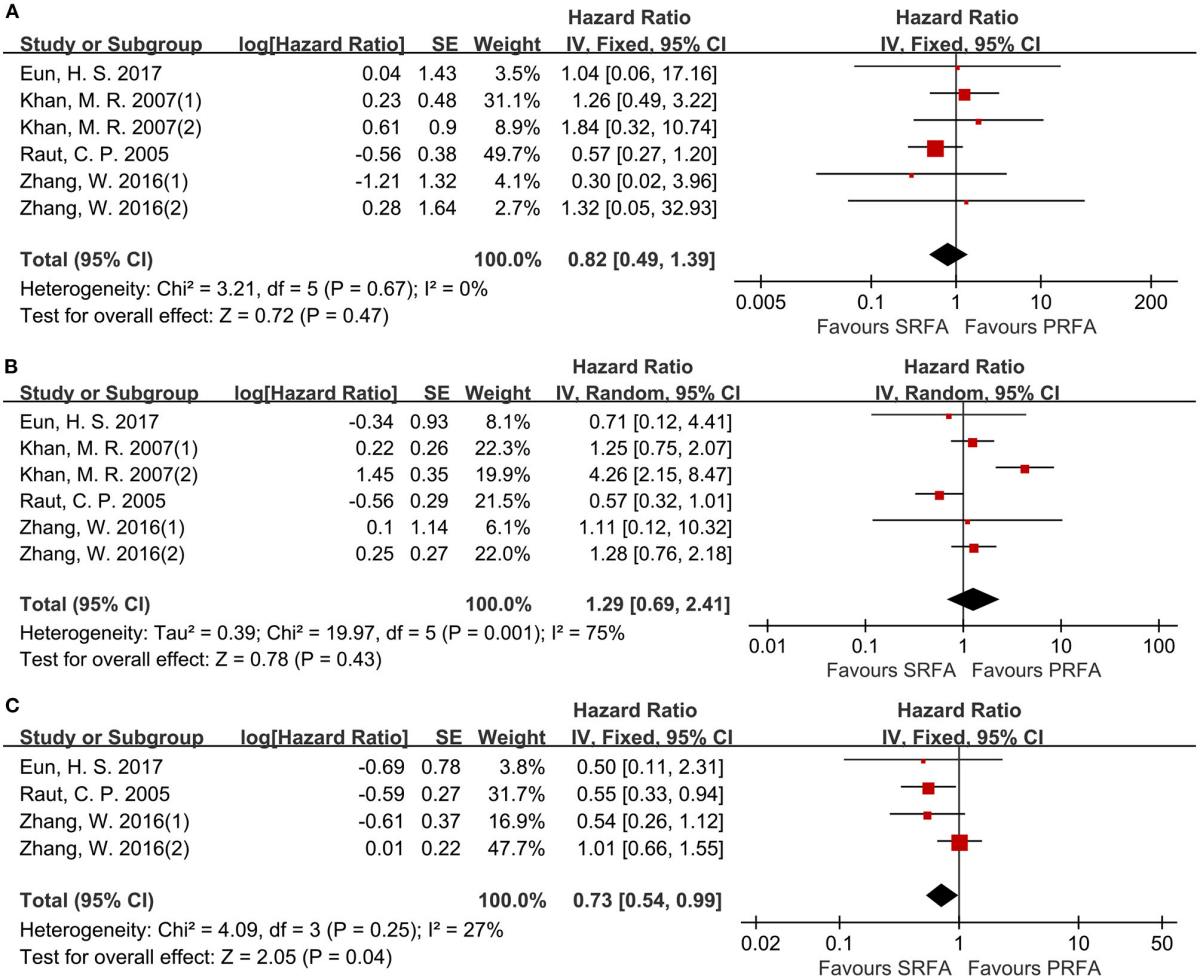
Forest plot for DFS. **(A)** Forest plot for 1-year DFS indicates no significantly between the PRFA group and the SRFA group (HR, 0.82; 95% CI: 0.49–1.39). **(B)** Forest plot for 3-year DFS indicates no significantly between the PRFA group and the SRFA group (HR, 1.29; 95% CI: 0.69–2.41). **(C)** Forest plot for 5-year DFS indicates the PRFA group was significantly lower than the SRFA group (HR, 0.73; 95% CI: 0.54–0.99).

Data on disease-free survival (DFS) were reported in six cohorts (11, 17, 20, 21). For the 1- and 3-year DFS, there was no difference between the PRFA and the SRFA groups (Figures 7A,B), with HR of 0.82 (95%CI 0.49–1.39) and 1.29 (95%CI 0.69–2.41), respectively. The 5-year DFS of the PRFA group was significantly lower than the SRFA group, with an HR of 0.73 (95%CI 0.54–0.99) and mild heterogeneity (*I*^2^ = 27%, *p* = 0.25; Figure 7C).

### Sensitivity Analysis and Publication Bias

The sensitivity analysis included eight retrospective studies that scored eight or more stars on the mNOS. There was no significant change in complete ablation rate, complication, total recurrence, intrahepatic recurrence, and extrahepatic metastasis. For local recurrence, the degree of between-study heterogeneity significantly decreased and there was significantly less recurrence in the SRFA group.

**Figure 8:**
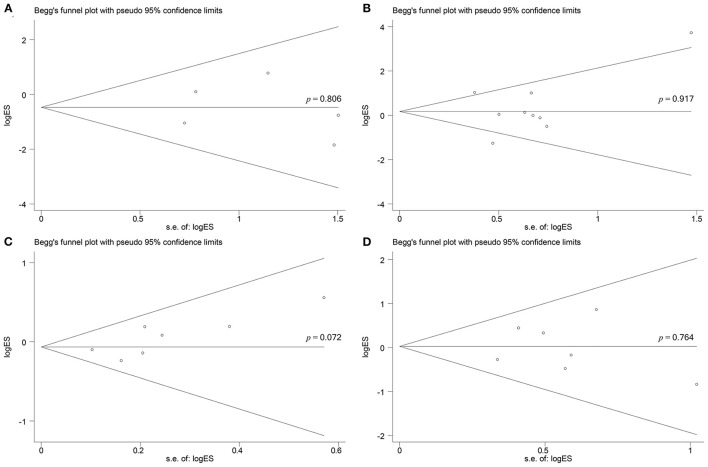
Begg's test for does not indicate any evidence of publication bias. **(A)** Begg's funnel plot with pseudo 95% confidence limits in complete ablation rate (*p* = 0.806). **(B)** Begg's funnel plot with pseudo 95% confidence limits in complications (*p* = 0.917). **(C)** Begg's funnel plot with pseudo 95% confidence limits in total recurrence rate (*p* = 0.072). **(D)** Begg's funnel plot with pseudo 95% confidence limits in intrahepatic recurrence rate (*p* = 0.764).

According to the Begg's rank correlation test, there was no significant difference in publication bias in complete ablation rate (*p* = 0.806; Figure 8A), complication (*p* = 0.917; Figure 8B), total recurrence rate (*p* = 0.072; Figure 8C), and intrahepatic recurrence rate (*p* = 0.764; Figure 8D), respectively.

## Discussion

Radiofrequency ablation has emerged as an important alternative treatment to surgery for HCC (23). In this study, there were no significant differences in complete ablation rate, total tumor recurrence rate, and perioperative complications between the PRFA and SRFA groups. In the sensitivity analysis, the PRFA group had a significantly higher local recurrence rate compared to the SRFA group.

There is a consensus that tumor size is an important risk factor for local recurrence, with a meta-analysis of 34 studies revealing that maximum benefit was observed when the tumor diameter of HCC was less than 2 cm (24). A higher local recurrence rate for a larger size of HCC could be due to several factors. For large tumors, a large number of precisely calculated overlapping coagulations is necessary; statistical data showed that 14 overlapping coagulations are required to cover a 3 cm tumor and its safety margin with an electrode that produces perfect spherical coagulation of 3 cm (25). It is difficult to visualize the tumor after the first coagulation session due to the formation of a hyperechogenic microbubble cloud using ETUS and IOUS. Unfortunately, when more than one treatment session is needed to achieve complete ablation, there is a higher risk of local recurrence (26). A third factor is that larger tumors have irregular borders more frequently than small tumors, making it hard to achieve an oncologic safety margin. According to the hepatectomy principle, the required minimum length of safety margin is 5.5 and 6 mm to achieve 99% and 100% micrometastasis clearance, respectively, in surrounding the liver of HCC patients (27). In our meta-analyses, the tumor size in the PRFA group was smaller than in the SRFA group. However, the PRFA group appeared to have a lower complete ablation rate than the SRFA group, indicating that RFA through a surgical approach may achieve a more precise and complete ablation, particularly in larger tumor nodules.

The pattern of tumor recurrences, such as the total recurrence rate and extrahepatic metastasis, did not differ between PRFA and SRFA. However, sensitivity analysis revealed that the local recurrence rate was higher in the PRFA group compared to the SRFA group. Although the morbidity of malignant seeding in the needle tract is low, two malignant seeding procedure-related cases occurred only in PRFA, making it difficult to avoid (28).

The tumor location has a significant influence on local tumor control (29). The difficulty in inserting the electrode, as well as in obtaining a sufficient ablative margin along with the liver capsule, have previously been attributed to PRFA for subcapsular tumors (30). In addition, perivascular tumor location has been identified as another risk factor for local tumor recurrence after RFA, mainly due to the insufficient ablative margin created by RFA due to the heat-sink effect (31). The surgical approach, which is different from PRFA, has several advantages: the IOUS probe is placed directly on the liver surface, without sound attenuation by the skin and subcutaneous tissue. Several studies have reported a 30% increase in tumor detection rate using intraoperative ultrasound (32–34). Improved visibility not only allows for more accurate insertion of electrodes and an increased possibility of completely covering the tumor, including its irregular margins, satellites, and safety margin but also prevents damage to organs during the procedure (35). Furthermore, the acoustic window is much wider compared to external ultrasound, which is hampered by the interposition of the ribs and bowel (36). In cases where overlapping coagulations are necessary, the surgical route allows for multiple parallel reinsertions of the electrode, which is difficult to achieve percutaneously. The open approach allows for a larger degree of freedom when inserting the electrodes at an optimal angle, with mobilization of the liver if necessary (37, 38), and ablation zone enlargement can be achieved by using the Pringle maneuver to reduce liver blood flow by 30–40% (39). Because of the pneumoperitoneum and the upward movement of the diaphragm, liver movement is minimal, allowing for precise electrode placement using the laparoscopic approach (17, 40).

The major complication rate of PRFA and SRFA remains controversial. The surgical approach is more invasive and has a significantly higher rate of postoperative ascites than PRFA. Although the incidence of more severe complications, such as bile duct injury, liver abscess, and procedure-related hemorrhage, appeared to be lower in patients than in PRFA, the results were not statistically significant. Therefore, skin burn would not occur due to the real-time visual ablation.

The advantages of PRFA include less invasiveness and a shorter hospital stay (10, 20). Interestingly, a meta-analysis found that there was no significant difference in the duration of hospital stay. However, after removing several studies using the laparoscopic approach (18, 22), it was discovered that PRFA had a shorter postoperative hospital stay than SRFA.

In the SRFA group, less recurrence may lead to a significant difference in DFS; our meta-analysis showed that the 5-year DFS of the SRFA group was significantly higher than the PRFA group. However, there was no significant difference in the 1-, 3-, and 5-year OS rates. Based on the current information, it is difficult to come up with a convincing explanation for this difference. This could be due to the difference in the follow-up treatment after radical ablation (10, 20), requiring a large sample size and more comprehensive follow-up data for follow-up evaluation, instead of the RFA approach.

This review has several limitations. First, all included studies were observational, with a lack of randomized data available, resulting in a selection bias. Second, most of the included studies were conducted over different periods and with different ablation devices. The evolving ablation technology and ultrasonic experience affected the accuracy of ablation. Third, it is difficult to compare PRFA and SRFA in terms of tumor location in the liver; for example, RFA using a surgical approach allows easy access to tumors located in the superior right lobe of the liver, which are often hard to reach percutaneously. In addition, variations in the studied populations may influence the patients' outcomes. Furthermore, the size of the cohort samples was relatively small, leading to a reduction in the quality of the conclusions, while the quality of the studies included, which were assessed using the NOS, was moderate. This is, to our knowledge, the first meta-analysis that attempted to determine the superiority of SRFA over open and laparoscopic approaches in patients with HCC. Therefore, we can reasonably conclude that SRFA provides superior local control and should be the first choice for any patient who can tolerate laparoscopy or laparotomy. Further studies with randomized trials are required to validate the results of this study.

## Conclusion

In conclusion, based on the findings of this study, the surgical route is the preferred approach for RFA, as it resulted in a better local control rate and disease-free survival.

## Data Availability Statement

The datasets presented in this study can be found in online repositories. The names of the repository/repositories and accession number(s) can be found in the article/Supplementary Material.

## Author Contributions

XY, CW, and ZH contributed to the screening and data collection. TM and ZH contributed to the assessment of the included article. XB and LX contributed to the data analysis. XH and YL contributed to the writing of the manuscript. XC contributed to provide expert insight into the revision of the manuscript and being as corresponding author. All authors approved the final version of the reports and contributed to the study concept, design, data interpretation, and discussion.

## Funding

This research supported by Sanming Project of Medicine in Shenzhen (Nos. SZSM202011010 and SZSM201812079) and Shenzhen High-level Hospital Construction Fund.

## Conflict of Interest

The authors declare that the research was conducted in the absence of any commercial or financial relationships that could be construed as a potential conflict of interest.

## Publisher's Note

All claims expressed in this article are solely those of the authors and do not necessarily represent those of their affiliated organizations, or those of the publisher, the editors and the reviewers. Any product that may be evaluated in this article, or claim that may be made by its manufacturer, is not guaranteed or endorsed by the publisher.
