# Supplementary figures and images for: Meta-analysis of Percutaneous vs. Surgical Approaches Radiofrequency Ablation in Hepatocellular Carcinoma
Source: Front Surg. 2022 Jan 4;8:788771. doi: 10.3389/fsurg.2021.788771 (PMC8763842; doi:10.3389/fsurg.2021.788771)

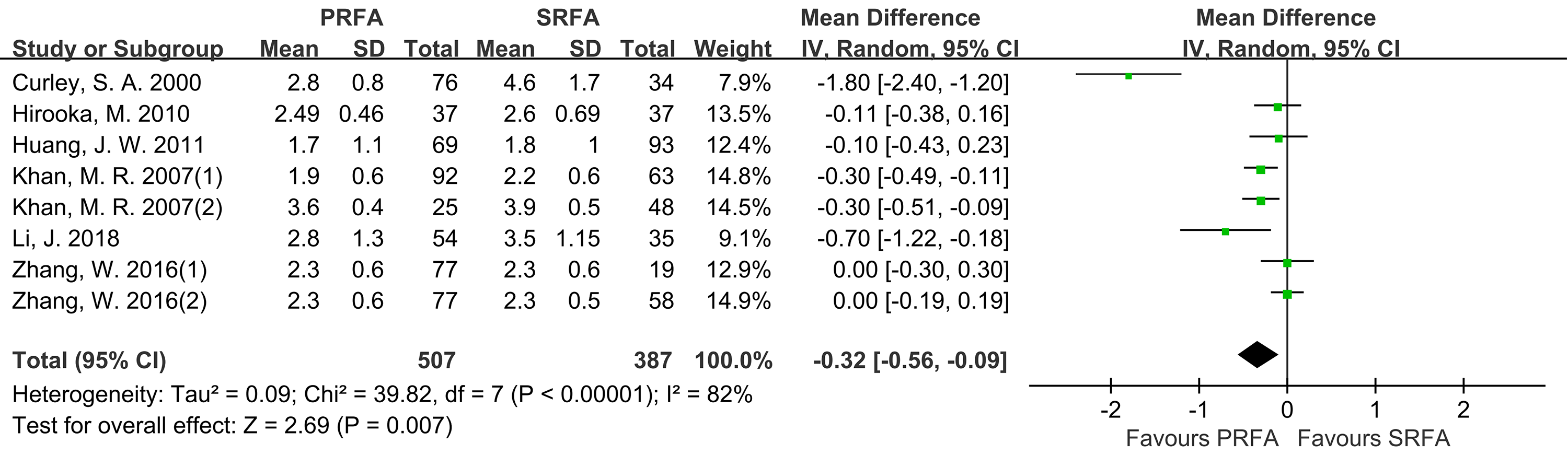

Supplement: Supplementary file 1 [file Image_1.TIF]

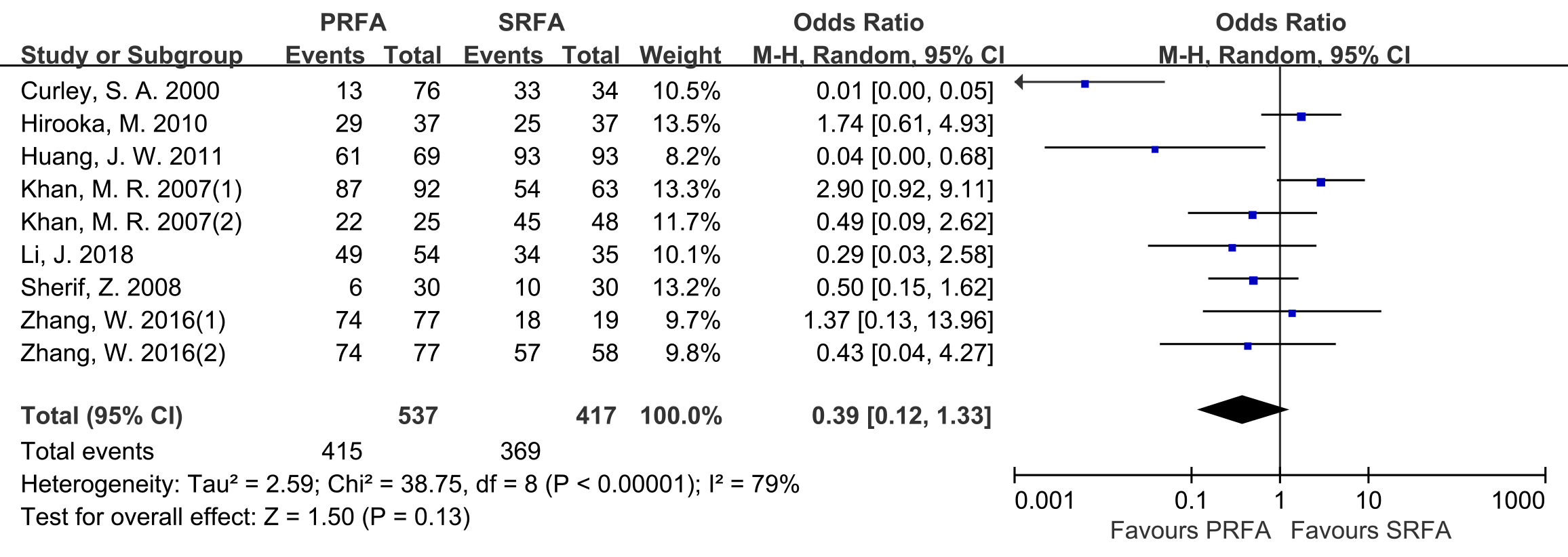

Supplement: Supplementary file 2 [file Image_2.TIF]

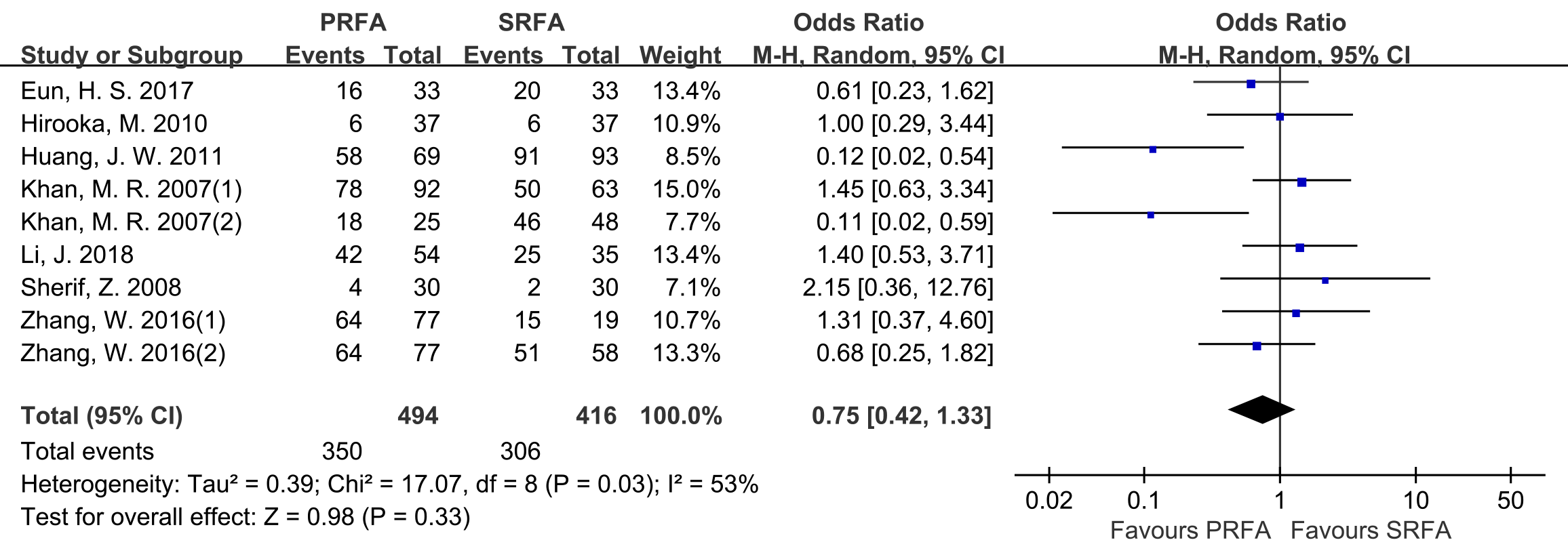

Supplement: Supplementary file 3 [file Image_3.TIF]
